# Comparison of five different methodologies for evaluating ankle–foot orthosis stiffness

**DOI:** 10.1186/s12984-023-01126-7

**Published:** 2023-01-22

**Authors:** Benjamin R. Shuman, Deema Totah, Deanna H. Gates, Fan Gao, Andrew J. Ries, Elizabeth Russell Esposito

**Affiliations:** 1 Center for Limb Loss and Mobility, VA Puget Sound, 1660 S Columbian Way, Seattle, WA USA; 2 The Henry M. Jackson Foundation for the Advancement of Military Medicine, Inc., Bethesda, MD USA; 3 Department of Mechanical Engineering, University of Iowa, Iowa City, IA USA; 4 School of Kinesiology, University of Michigan, Ann Arbor, MI USA; 5 Department of Kinesiology and Health Promotion, University of Kentucky, Lexington, KY USA; 6 James R. Gage Center for Gait & Motion Analysis, Gillette Children’s Specialty Healthcare, St. Paul, MN USA; 7 DOD-VA Extremity Trauma and Amputation Center of Excellence (EACE), Joint Base San Antonio Fort Sam Houston, TX USA

**Keywords:** Rotational stiffness, Mechanical testing, Bench testing, Methods comparison, Carbon fiber, Stiffness measurement, Testing standards, Exoskeleton

## Abstract

**Background:**

The mechanical properties of an ankle–foot orthosis (AFO) play an important role in the gait mechanics of the end user. However, testing methodologies for evaluating these mechanical properties are not standardized. The purpose of this study was to compare five different evaluation frameworks to assess AFO stiffness.

**Method:**

The same 13 carbon composite AFOs were tested with five different methods. Four previously reported custom test fixtures (the BRUCE, KST, SMApp, and EMPIRE) rotated an AFO into dorsiflexion about a defined axis in the sagittal plane. The fifth method involved quasi-static deflection of AFOs into dorsiflexion by hanging weights (HW) from the footplate. AFO rotational stiffness was calculated as the linear fit of the AFO resistive torque and angular deflection. Differences between methods were assessed using descriptive statistics and a repeated measures Friedman with post-hoc Bonferroni–Holm adjusted Wilcoxon signed-rank tests.

**Results:**

There were significant differences in measured AFO stiffnesses between test methods. Specifically, the BRUCE and HW methods measured lower stiffness than both the EMPIRE and the KST. Stiffnesses measured by the SMApp were not significantly different than any test method. Stiffnesses were lowest in the HW method, where motion was not constrained to a single plane. The median difference in absolute AFO stiffness across methods was 1.03 Nm/deg with a range of [0.40 to 2.35] Nm/deg. The median relative percent difference, measured as the range of measured stiffness from the five methods over the average measured stiffness was 62% [range 13% to 156%]. When the HW method was excluded, the four previously reported test fixtures produced a median difference in absolute AFO stiffness of 0.52 [range 0.38 to 2.17] Nm/deg with a relative percent difference between the methods of 27% [range 13% to 89%].

**Conclusions:**

This study demonstrates the importance of developing mechanical testing standards, similar to those that exist for lower limb prosthetics. Lacking standardization, differences in methodology can result in large differences in measured stiffness, particularly for different constraints on motion. Non-uniform measurement practices may limit the clinical utility of AFO stiffness as a metric in AFO prescription and future research.

**Supplementary Information:**

The online version contains supplementary material available at 10.1186/s12984-023-01126-7.

## Background

Ankle–foot orthoses (AFOs) are external braces used to support or augment the ankle joint during activities of daily living. A wide variety of custom and commercial AFOs are available [1] to accommodate each AFO user’s individual needs [2, 3]. Differences among AFOs are largely driven by their geometry and mechanical properties. As such, evaluating AFO mechanical properties has received increasing attention in the literature [1, 4, 5]. One of the more common mechanical properties evaluated is the rotational stiffness about the ankle joint, calculated as the change in resistive torque over the change in ankle angle [6–12].

Unlike prosthetics where testing standards have been established for prosthetic feet [13, 14] (e.g. ISO 10328 and ISO 22675), no such standards exist for AFOs. Some AFO manufacturers have attempted to adapt these prosthetic standards for AFOs [15, 16], but the field largely lacks consistent methodology. Methodological differences include inconsistencies with mounting, alignment/bending axis, range of motion, and testing speed [1, 4, 5]. Only one study has directly compared AFO properties with two devices across three AFOs [11]. While similar stiffnesses were found in that study, it is unclear whether those results are generalizable across a range of AFO designs and materials or to other testing devices.

Thus, the goal of this study was to compare the stiffnesses of carbon composite AFOs evaluated using methodologies that have been reported in the literature. The first four methods were previously reported test fixtures evaluated for repeatability: The Bi-articular Reciprocating Universal Compliance Estimator (BRUCE) [6], Kentucky Stiffness Tester (KST) [10], the AFO Stiffness Measurement Apparatus (SMApp) [11] and the device for Evaluating Mechanical Properties In Rotating Exoskeletons (EMPIRE) [12]. The fifth method manually measured the deflection of an AFO elicited from hanging weights off of the toe, similar to the approach used in previous studies [17–19].

## Methods

### AFOs tested

Thirteen commercially available carbon composite, non-articulated AFOs were tested including: the Blue Rocker, Blue Rocker 2 ½, ToeOFF, and ToeOFF 2 ½ from Allard (Helsingborg, Sweden), the WalkOn Reaction and WalkOn Reaction Plus from Ottobock (Duderstadt, Germany), the SpryStep, SpryStep Max and SpryStep Plus from Thuasne (Levallois-Perret, France), and the Matrix, Matrix Max, Matrix Max2 and Matrix Supermax from Trulife (Dublin, Ireland) (Additional file 1: Table S1). All AFOs except the Ottobock AFOs were the same specimens as previously reported in Shuman & Russell Esposito [12]. All AFOs were right foot models, size large. The same AFO specimens were independently evaluated by each methodology.

### Testing methods

**Fig. 1:**
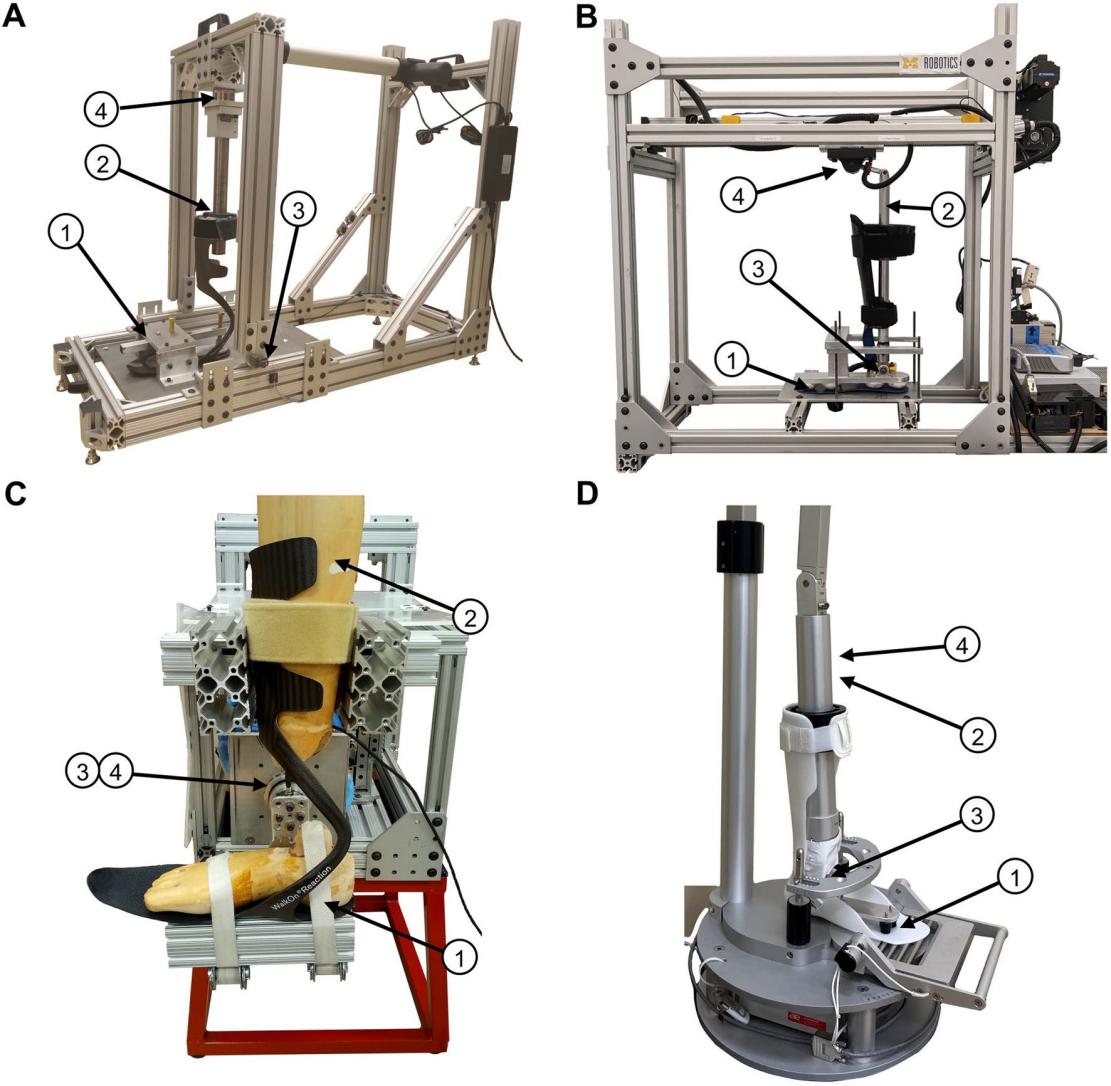
Overview of existing test fixtures. **A** EMPIRE, **B** SMApp, **C** KST, and **D** BRUCE. All fixtures secure the AFO for testing by clamping down the footplate using a mock/surrogate/test foot (1) and strapping the tibial cuff to a surrogate shank (2). Angular displacment of the AFO is measured at the axis of rotation using an encoder (3) and applied loads are measured using a load cell (4). Note that the AFO pictured in the BRUCE was not one tested in this study

**Fig. 2:**
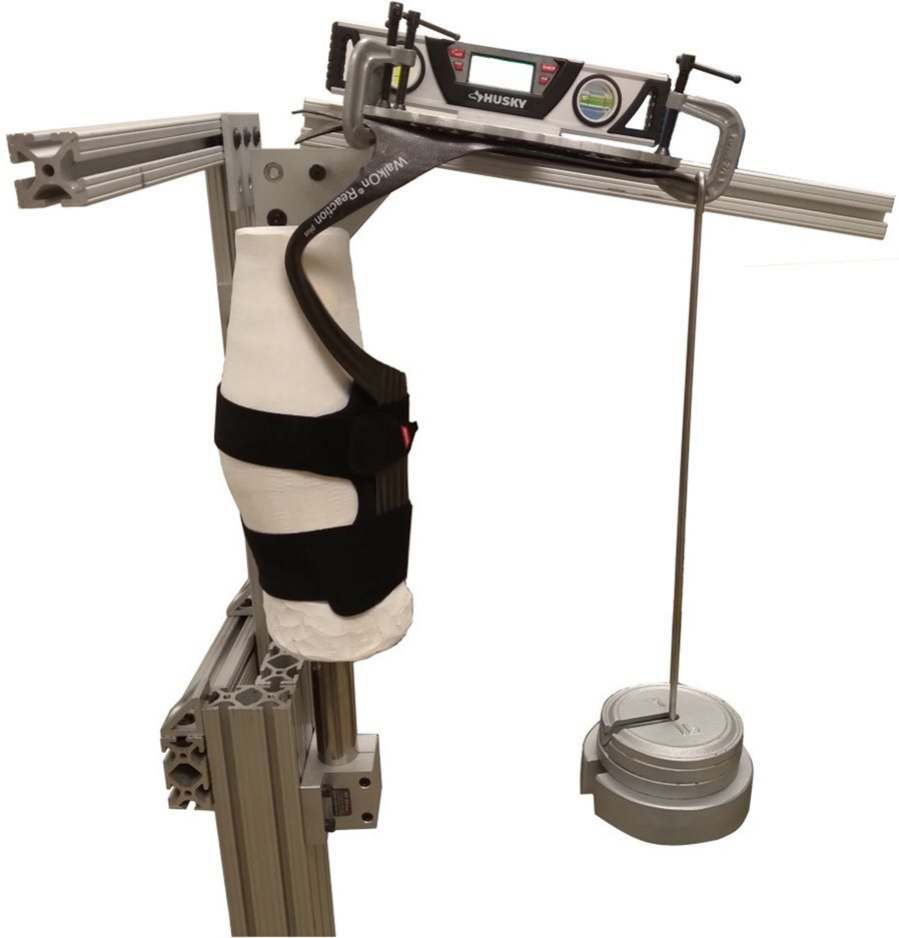
Setup for hanging weight method. In this approach, an AFO is strapped to a surrogate shank and the footplate is clamped to a backing plate. Weights are hung from the AFO toe and the angle of deflection is measured with an inclinometer. Torque is computed from the applied load and the moment arm measured to the center of the surrogate shank

Four previously reported test fixtures were used to evaluate AFO stiffness (Fig. 1). Common to all four designs, an AFO was rotated about a fixed axis representing the ankle joint. Methodological differences across test fixtures are summarized in Table 1. Further detail on the design of each test fixture can be found in previous publications (BRUCE [6], KST [10], SMApp [11], and EMPIRE [12]). We also used a simple method to quasi-statically load an AFO by manually measuring deflections from known loads (see ‘Hanging weights method’, Fig. 2).

**Table 1:** Comparison of fixture designs

Fixture name	EMPIRE	SMApp	KST	BRUCE
**Fixture capabilities**				
Maximum Testable Dorsiflexion	25 deg	25 deg	20 deg	20 deg
Maximum Testable Plantarflexion	5 deg	25 deg	30 deg	10 deg
Tested Rotational Speed	0.75 deg/s	1 deg/s	0.75 deg/s	Manual
Fixture Speed Range	0.75 deg/s	0 to 100 deg/s	0 to 30 deg/s	Manual
Load Measurement	Six Axis load cell	Uniaxial Force Transducer	Uniaxial Torque Sensor	Uniaxial Force Transducer
Max Ankle Torque	89 Nm †	626 Nm	25 Nm	133.5 Nm
Sample rate	100 Hz	35 Hz	1000 Hz	100 Hz
**AFO Mounting and Alignment**				
Footplate Attachment	Fixed/Clamped	Fixed/Clamped	Fixed/Clamped	Fixed/Clamped
Tibial Cuff Attachment	Fixed/Clamped	Mounted to a linear bearing	Fixed/Clamped	Mounted to a linear bearing
Transverse Alignment	Medial Border	Bisected	Medial Border	Bisected
Axis of Rotation Vertical Position	81 mm	55 mm^‡^	81 mm	75 mm
Axis of Rotation Fore/Aft Position	Uncontrolled, based on surrogate shank fit	Uncontrolled, based on surrogate shank fit	Uncontrolled, based on surrogate shank fit	Uncontrolled, based on surrogate shank fit
**Data processing**				
AFO Precycling	1st cycle discarded	Precycled once prior to recorded testing	1st cycle discarded	Precycled prior to recorded testing
Adjusted for fixture inertia?	Yes	Yes	No	No
Initial data Filtering	1 Hz LP	No	No	10 Hz LP

^†^Value reported for the configuration used in this testing. Maximum testing torque at the ankle is dependent upon AFO geometry (Shuman 2021)

^‡^Physical rotational axis is located at 55 mm (Totah 2021). Data were computationally adjusted to match the 81 mm rotation height in the EMPIRE

### AFO mounting and rotation axis in existing test fixtures

All footplates were secured to the testing fixture to minimize foot motion contributions to stiffness. A surrogate shank was securely strapped to the AFO shell in all test fixtures. The BRUCE and SMApp permitted sliding (pistoning) of the AFO in the attachment to the surrogate shank via a linear bearing, whereas the KST and EMPIRE were strapped to minimize AFO motion relative to the surrogate shank. Surrogate shank diameters used in this study were 100 mm on the BRUCE, 100 mm (largest portion) on the KST, and 115 mm on the EMPIRE, while the SMApp made use of two best fit surrogate shanks with tapered diameters ranging between 90 and 130 mm. Each system has an adjustable axis (height) of rotation from the footplate. The BRUCE was adjusted to 75 mm through the use of several sizes of mock feet. The KST and EMPIRE fixtures were adjusted using slots to 81 mm. The SMApp rotation height is 55 mm and computationally adjusted to match a specified axis (81 mm in this study). Transverse plane alignment varied by test fixture. In the EMPIRE and KST the direction of progression was aligned along the medial border of the AFO; the BRUCE and SMApp aligned the direction of progression along a line bisecting the medial and lateral border of the AFO.

### Data collection and processing

For consistency between test fixtures, in each testing session an AFO was first deflected into 3 to 5 degrees of plantarflexion and then deflected into dorsiflexion. Maximum dorsiflexion angle was 20 degrees or as limited by the fixture geometry or load cell capacities. The rotational speeds were closely matched in the automated test fixtures (KST: 0.75 deg/s; SMApp 1 deg/s; EMPIRE: 0.75 deg/s). The BRUCE was manually rotated. The number of cycles within a session and number of test sessions varied by AFO and fixture (Additional file 2: Table S2). Different operators collected data on each fixture, with a single operator for the SMApp, BRUCE and KST, and two operators on the EMPIRE [12]. Between each testing session the AFO was removed from the fixture.

AFOs were pre-cycled in the SMApp and BRUCE fixtures to ensure the AFO was well seated in the test fixture and to stabilize the stiffness measurements. The first cycle in each testing session of the EMPIRE and the KST were treated as a pre-conditioning cycles and discarded from further analysis.

All test fixtures simultaneously collected load cell and angular position data between 35 and 1000 Hz. In accordance with prior literature describing the test methodologies, raw data collected on the KST were not filtered while data collected on the BRUCE and EMPIRE were low-pass filtered at 10 and 1 Hz, respectively [6, 12]. In the SMApp, any signal artifacts were identified as sample-to-sample variations greater than 10 deg or 10 N (corresponding to about 350 deg/s or N/s slope) and removed from the raw time signals [11]. For the SMApp and EMPIRE, we applied calibration curves to adjust the measured load cell data to account for the gravitational loads and mechanical losses of the test fixture. For consistency across all testing devices, AFO stiffnesses were computed as the linear fit of the torque angle curve for each cycle while moving into dorsiflexion [12]. To avoid periods of acceleration and deceleration when transitioning between plantarflexion and dorsiflexion, the stiffness was calculated over a smaller range of deflection than the total range of motion (Additional file 2: Table S2) [11, 12]. For those AFOs tested at the full range of motion, AFO stiffness was computed between 0 and 18 degrees of dorsiflexion.

### Hanging weights method

A fifth method incrementally deflected an AFO into dorsiflexion by hanging a weight (HW) from the end of the footplate (Fig. 2). The AFO was mounted by strapping the tibial cuff to an inverted plaster surrogate shank. Similar to the above test fixtures, deflection was constrained to the AFO strut by clamping an aluminum plate to the AFO footplate. The AFO was aligned such that the rotating frame was parallel to the medial border of the AFO. Since the deflection to the AFO was not constrained to a specific axis as in the other test fixtures, the height of the rotating frame was adjusted as needed such that the measurement surface was aligned to the footplate. The AFOs were loaded with an increment of 0.45 kg up to a maximum 6.80 kg. Two loadings were performed for each AFO, by a single operator. For each loading, the moment arm was measured as the perpendicular distance between the center of the surrogate shank and the line of load application. To consistently measure deflection in a uniform plane and avoid additional loads from the weight of the inclinometer, the inclinometer was mounted to a rotating frame. Out of plane deflections were not measured.

### Statistical analysis

For each method, AFO stiffnesses were computed as the linear fit of the torque angle curve for each cycle. The overall average stiffness was computed for each AFO in each test method across all measured cycles. We compared the differences in AFO stiffness across the test methods using a Friedman test in MATLAB (The MathWorks Inc., Natick, MA) with α = 0.05. Post-hoc comparisons between methods were computed using Wilcoxon signed-rank tests with a Bonferroni-Holm correction for 10 between-device comparisons [20]. Differences in AFO stiffness across test methods were measured using descriptive statistics (median and range). Because the AFOs included span a range of stiffnesses, we report the relative percent differences in AFO stiffness across the test methods, computed as the range in measured stiffness divided by the average stiffness across all methods. Differences in stiffness for each AFO between each pair of test methods were measured using descriptive statistics (median and IQR) [21].

For the previously reported fixtures, we also computed the average stiffness for each AFO test session in each test method across all measured cycles. For each fixture we compared the differences in AFO stiffness across test sessions using descriptive statistics (median and range). Because the AFOs included span a range of stiffnesses, we report the relative percent differences in AFO stiffness across the sessions, computed as the range in measured stiffness divided by the average stiffness. For each AFO the ratio of the range in stiffness across fixtures to the largest intersession range of stiffness was computed.

## Results

All stiffness devices measured a linear relationship between torque and displacement (r^2^ > 0.95) for all trials (Additional file 3: Fig. S1, Additional file 4: Fig. S2, Additional file 5: Fig. S3, Additional file 6: Fig. S4, Additional file 7: Fig. S5, Additional file 8: Fig. S6, Additional file 9: Fig. S7, Additional file 10: Fig. S8, Additional file 11: Fig. S9, Additional file 12: Fig. S10, Additional file 13: Fig. S11, Additional file 14: Fig. S12, Additional file 15: Fig. S13, panel A).

**Fig. 3:**
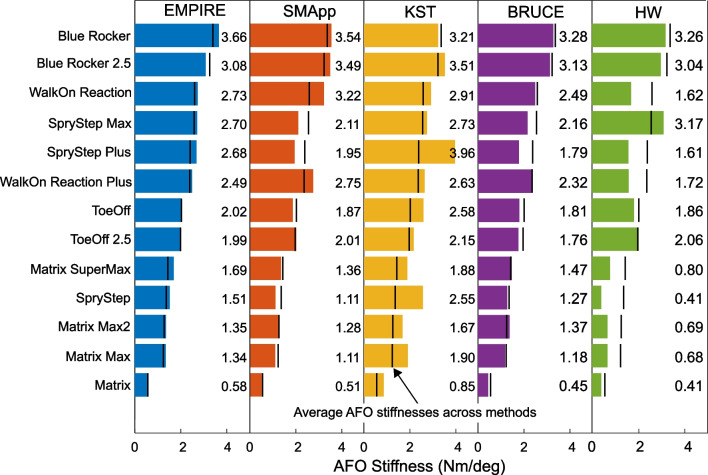
Measured AFO stiffnesses for each of the five test methods. AFOs are ordered from the largest average stiffness to the smallest average stiffness. Average stiffnesses across methods are indicated by an overlaid black line

Each method identified the Blue rocker as the stiffest AFO (except the KST, which ranked it third stiffest of 13), with stiffnesses ranging from 3.21 Nm/deg (KST) to 3.66 Nm/deg (EMPIRE) (Fig. 3). All test methods identified the Matrix as the least stiff (0.41 Nm/deg, HW to 0.85 Nm/deg, KST). Across all AFOs, the median difference in absolute AFO stiffness between methods was 1.06 Nm/deg (range: 0.40 to 2.35 Nm/deg). The relative percent differences in stiffnesses was a median of 62% of the average stiffness across methods (range: 13% in the Blue Rocker to 156% in the SpryStep). With the HW method excluded, the median difference in absolute AFO stiffness between methods decreased to 0.52 Nm/deg (range: 0.38 to 2.17 Nm/deg) with relative percent differences in stiffnesses a median of 27% of the average stiffness across methods (range: 13% in the Blue Rocker to 89% in the SpryStep).

**Fig. 4:**
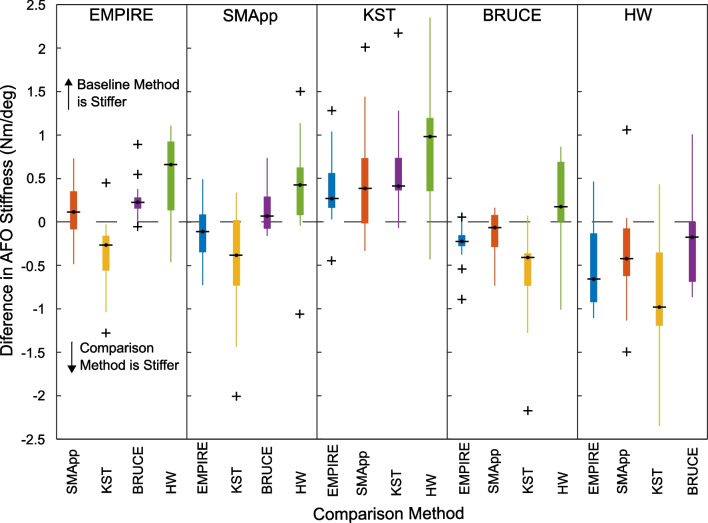
Box plot of differences in measured AFO stiffness between test methods. Each method is used as a baseline and compared to each of the other test methods, where the comparison method is indicated by color (blue: EMPIRE, red: SMApp, yellow; KST, purple: BRUCE, green: HW). Positive values indiate that the baseline method is stiffer than the comparison method. The KST measured stiffnesses are larger than any of the other test fixtures while the HW measured the lowest stiffnesses

Measured AFO stiffness was different across the test methods (p < 0.001) using a Friedman Test. The HW method produced the lowest stiffness values of any method, which were a median of 0.66 (IQR: 0.13 to 0.94), 0.42 (0.08 to 0.62), 0.91 (0.35 to 1.24), and 0.18 (0.01 to 0.67) Nm/deg lower than the EMPIRE, SMApp, KST, and BRUCE respectively (Fig. 4). Statistically, the HW method was significantly lower than the EMPIRE (p = 0.006 < 0.007 = α/7, Wilcoxon signed-rank post-hoc), and KST (p = 0.002 < 0.006 = α/8) but did not reach the corrected level of significance for the SMApp (p = 0.027 > 0.013 = α/4) or the BRUCE (p = 0.11 > 0.017 = α/3). The KST method measured stiffnesses were significantly greater than the BRUCE (p < 0.001 < 0.005 = α/10) and the EMIRE (p = 0.008 = 0.008 = α/6) by a median of 0.41 (0.36 to 0.74) and 0.27 (0.16 to 0.56) Nm/deg respectively, but not significantly greater than the SMApp (p = 0.013 > 0.01 = α/5) by a median of 0.38 (-0.02 to 0.73) Nm/deg. Across the tested AFOs, the EMPIRE was not significantly different than the SMApp (p = 0.27 > 0.05 = α) by a median of 0.11 (− 0.08 to 0.35) Nm/deg, and was stiffer than the BRUCE (p = 0.001 < 0.006 = α/9) by a median of 0.22 (0.15 to 0.28) Nm/deg. The best agreement between any two fixtures’ median values was between the SMApp and the BRUCE with the SMApp not significantly different (p = 0.13 > 0.025 = α/2) with a median of 0.07 (− 0.08 to 0.29) Nm/deg.

Within each previously reported test fixture, median intersession ranges in stiffness were 0.27 Nm/deg in the Empire, 0.11 Nm/deg in the SMApp, 0.11 Nm/deg in the KST, and 0.03 Nm/deg in the BRUCE, (Additional file 3: Fig. S1, Additional file 4: Fig. S2, Additional file 5: Fig. S3, Additional file 6: Fig. S4, Additional file 7: Fig. S5, Additional file 8: Fig. S6, Additional file 9: Fig. S7, Additional file 10: Fig. S8, Additional file 11: Fig. S9, Additional file 12: Fig. S10, Additional file 13: Fig. S11, Additional file 14: Fig. S12, Additional file 15: Fig. S13, panel B). Median relative percent differences in stiffness across sessions were 10% (range: 2 to 54%), 3% (< 1 to 14%), 5% (2 to 38%), and 1% (< 1 to 3%) for the EMPIRE, SMapp, KST, and BRUCE respectively. Differences in average measured stiffnesses across fixtures were a median of 2.06 (range: 0.97 to 5.60) times greater than the largest intersession differences for each AFO. Only the intersession differences in stiffness of the Matrix Max 2 (tested in the EMPIRE) were larger than the differences between fixtures.

## Discussion

This study demonstrates significant differences in the calculated rotational stiffness of AFOs among five test methods. The BRUCE, KST, SMApp, and EMPIRE are all conceptually similar in their design and operation, constraining the rotation of an AFO about a single axis and deflection to the strut. While not all methods were significantly different from one another, these four previously reported test methods, still had a median inter-method difference of 27% of the average measured stiffness.

A hanging weight (HW) method was included for its simplicity and similarity to previous reports in the literature [17–19], but was the most different from the other methods in design and produced the lowest measured stiffnesses. Unlike the other methods, the HW method imparted minimal constraints on the AFO’s motion, resulting in a shifting center point of rotation. The lack of constraints in the HW method allowed the AFOs to experience out of plane rotations, without regard for anatomical motion. While neither the height of rotation, nor the out of plane rotations were quantified in this study, AFOs with lateral struts deflected into inversion and external rotation in addition to dorsiflexion, while AFOs with medial struts also deflected into eversion and internal rotation. These additional deflections may have contributed to the consistently lower stiffnesses measured by the HW method, especially for the less rigid AFOs. With the other four custom fixtures, three considerations were incorporated for consistency. First, AFO stiffnesses may be sensitive to rotational speed [22–24], thus, all test fixtures were operated at relatively slow speeds except the BRUCE which is manually controlled. Second, the heights of the rotation axes were either mechanically or numerically adjusted to be similar (75–81 mm), as rotation axis may impact stiffness [10, 25]. Third, stiffnesses were all computed as the linear fit of the torque–angle curve while loading into dorsiflexion as previous work with these fixtures have computed stiffness differently, using quasi-static positions [10], the torque–angle during loading [11, 12], and the average-torque angle during loading and unloading [6].

## Limitations

Differences in test fixtures and alignment practices exist, all of which may impact the measured stiffnesses. While the height of the rotation axis was similar across the four custom test fixtures, the fore/aft position of the rotation axis was dependent on the geometry of the surrogate shank, which may have impacted measured AFO stiffness. Transverse alignments also varied between methods which may impact stiffness by changing the direction of load application [26, 27] or altering the axis of the rotation. Additional testing nuances, such as differences in the footplate clamping block, differences in the surrogate shank height, differences in total deflection, and whether the AFO surrogate shank was allowed to translate/rotate (as in the SMApp and the BRUCE) may have also contributed to inter-method differences in AFO stiffness. For example, while all fixtures clamped the footplate with the intent to constrain deflection to the strut, differences in the clamping configurations may have provided different movement constraints, allowing a portion the footplate to deform under load. Although the rotational speed was similar between the KST, EMPIRE, and SMApp (~ 1 deg/s), the BRUCE rotational speed varied by AFO and was between 14 and 50 deg/s, which may have contributed to lower measured AFO stiffnesses. An additional limitation was the different number of test session and cycles collected, which were based on the availability of the AFOs for testing and the methodologies for each fixture. For example, the KST cycled AFOs for 300 s rather than a pre-defined number of cycles. While the impact of each methodological difference remains unclear, this study demonstrates that the cumulative impact of these differences can have large impacts on measured AFO stiffnesses. It is important to note that these methodological differences do not make one method more or less accurate than another as differences in kinematics and anatomy between AFO users may also impact the user experienced stiffness.

However, it is also still important to identify factors that can lead to consistency and inconsistency in testing outputs. Prior work has suggested that AFO users may be sensitive to differences in stiffness as small as 12% [28]. Across the methods presented in this study we were only able to measure stiffness values that agreed within 15% of the average stiffness for 2 of the 13 AFOs tested, suggesting that if the goal of using stiffnesses to target AFO prescription is to be realized, uniform testing standards must be developed and adopted.

## Conclusions

Quantitative measurements of the mechanical properties of AFOs promise to improve the prescription of AFOs by enabling direct comparisons across models and manufactures. However, this study highlights factors that may contribute to differences in testing outputs and demonstrates the fundamental importance of developing uniform testing standards, similar to those that exist for lower limb prosthetics. Lacking standardization, differences in fixturing and alignment practices may result in large differences in measured stiffness, limiting the potential clinical utility.

## Supplementary Information

**Additional file 1: Table S1.** Manufacturer listed AFO details (size large).

**Additional file 2: Table S2.** Test parameters for each AFO by test method.

**Additional file 3: Figure S1.** Blue Rocker. A) Representative test session for each previously described test fixture. AFO stiffness is computed from the linear fit while the AFO is being loaded in dorsiflexion. B) Average stiffness across cycles for each test session for each of the previously described test fixtures.

**Additional file 4: Figure S2.** Blue Rocker 2.5. A) Representative test session for each previously described test fixture. AFO stiffness is computed from the linear fit while the AFO is being loaded in dorsiflexion. B) Average stiffness across cycles for each test session for each of the previously described test fixtures.

**Additional file 5: Figure S3.** ToeOff. A) Representative test session for each previously described test fixture. AFO stiffness is computed from the linear fit while the AFO is being loaded in dorsiflexion. B) Average stiffness across cycles for each test session for each of the previously described test fixtures.

**Additional file 6: Figure S4.** ToeOff 2.5. A) Representative test session for each previously described test fixture. AFO stiffness is computed from the linear fit while the AFO is being loaded in dorsiflexion. B) Average stiffness across cycles for each test session for each of the previously described test fixtures.

**Additional file 7: Figure S5.** WalkOn Reaction. A) Representative test session for each previously described test fixture. AFO stiffness is computed from the linear fit while the AFO is being loaded in dorsiflexion. B) Average stiffness across cycles for each test session for each of the previously described test fixtures.

**Additional file 8: Figure S6.** WalkOn Reaction Plus. A) Representative test session for each previously described test fixture. AFO stiffness is computed from the linear fit while the AFO is being loaded in dorsiflexion. B) Average stiffness across cycles for each test session for each of the previously described test fixtures.

**Additional file 9: Figure S7.** SpryStep. A) Representative test session for each previously described test fixture. AFO stiffness is computed from the linear fit while the AFO is being loaded in dorsiflexion. B) Average stiffness across cycles for each test session for each of the previously described test fixtures.

**Additional file 10: Figure S8.** SpryStep Max. A) Representative test session for each previously described test fixture. AFO stiffness is computed from the linear fit while the AFO is being loaded in dorsiflexion. B) Average stiffness across cycles for each test session for each of the previously described test fixtures.

**Additional file 11: Figure S9.** SpryStep Plus. A) Representative test session for each previously described test fixture. AFO stiffness is computed from the linear fit while the AFO is being loaded in dorsiflexion. B) Average stiffness across cycles for each test session for each of the previously described test fixtures.

**Additional file 12: Figure S10.** Matrix. A) Representative test session for each previously described test fixture. AFO stiffness is computed from the linear fit while the AFO is being loaded in dorsiflexion. B) Average stiffness across cycles for each test session for each of the previously described test fixtures.

**Additional file 13: Figure S11.** Matrix Max. A) Representative test session for each previously described test fixture. AFO stiffness is computed from the linear fit while the AFO is being loaded in dorsiflexion. B) Average stiffness across cycles for each test session for each of the previously described test fixtures.

**Additional file 14: Figure S12.** Matrix Max2. A) Representative test session for each previously described test fixture. AFO stiffness is computed from the linear fit while the AFO is being loaded in dorsiflexion. B) Average stiffness across cycles for each test session for each of the previously described test fixtures.

**Additional file 15: Figure S13.** Matrix SuperMax. A) Representative test session for each previously described test fixture. AFO stiffness is computed from the linear fit while the AFO is being loaded in dorsiflexion. B) Average stiffness across cycles for each test session for each of the previously described test fixtures.

## Data Availability

The datasets used and/or analyzed during the current study are available from the corresponding author on reasonable request.

## Abbreviations

EMPIRE: Evaluating Mechanical Properties in Rotating Exoskeletons
BRUCE: The Bi-articular Reciprocating Universal Compliance Estimator
SMApp: Stiffness Measurement Apparatus
KST: Kentucky Stiffness Tester
HW: Hanging Weights Test Method
IQR: Interquartile Range
AFO: Ankle Foot Orthosis

## Declarations

The view(s) expressed herein are those of the author(s) and do not reflect the official policy or position of the Henry M. Jackson Foundation for the Advancement of Military Medicine, Inc., the U.S. Army Medical Department, the U.S. Army Office of the Surgeon General the Department of the Army or the Department of Defense or the U.S. Government.
