# Supplementary material for: Comparison of five different methodologies for evaluating ankle–foot orthosis stiffness
Source: J Neuroeng Rehabil. 2023 Jan 22;20:11. doi: 10.1186/s12984-023-01126-7 (PMC9867850; doi:10.1186/s12984-023-01126-7)
Supplement: Supplementary file 1 — Additional file 1: Table S1. Manufacturer listed AFO details (size large). [file 12984_2023_1126_MOESM1_ESM.docx]

Supplimental Table 1: Manufacturer listed AFO details (size large)

| AFO | Manufacturer | Footplate Length | AFO Height |
| --- | --- | --- | --- |
|  |  |  |  |
| Blue Rocker | Allard | 270 mm | 432 mm |
| Blue Rocker 2.5 | Allard | 270 mm | 432 mm |
| ToeOff 2.5 | Allard | 270 mm | 432 mm |
| ToeOff | Allard | 270 mm | 432 mm |
| WalkOn Reaction | Ottobock | 310 mm* | 376 mm |
| WalkOn Reaction Plus | Ottobock | 310 mm* | 376 mm |
| SprySytep Max | Thuasne | 254-273 mm | 419 mm |
| SprySytep Plus | Thuasne | 260-289 mm | 425 mm |
| SprySytep | Thuasne | 260-289 mm | 359 mm |
| Matrix SuperMax | Townsend | 249-290 mm | 432 - 457mm |
| Matrix Max 2 | Townsend | 259-292 mm | 432 - 457mm |
| Matrix Max | Townsend | 249-290 mm | 432 - 457mm |
| Matrix | Townsend | 249-290 mm | 432 - 457mm |
| * Not reported by the manufacture, measured on the tested AFO | | | |
