# Supplementary material for: Comparison of five different methodologies for evaluating ankle–foot orthosis stiffness
Source: J Neuroeng Rehabil. 2023 Jan 22;20:11. doi: 10.1186/s12984-023-01126-7 (PMC9867850; doi:10.1186/s12984-023-01126-7)
Supplement: Supplementary file 2 — Additional file 2: Table S2. Test parameters for each AFO by test method. [file 12984_2023_1126_MOESM2_ESM.docx]

Supplimental Table 2: Test parameters for each AFO by test method.

|  | Sessions (total cycles) | | | | | | | | | | Deflection into Dorsiflexion (degrees) for stiffness calculation | | | | |
| --- | --- | --- | --- | --- | --- | --- | --- | --- | --- | --- | --- | --- | --- | --- | --- |
| AFO | EMPIRE | | KST | | BRUCE | | SMApp | | HW | | EMPIRE | KST | BRUCE | SMApp | HW |
| Blue Rocker | 4 | (8) | 2 | (19) | 3 | (13) | 2 | (6) | 2 | (2) | 18.0 | 8.0 | 8.0 | 18.0 | 5.3 |
| Blue Rocker 2.5 | 4 | (8) | 2 | (20) | 3 | (10) | 1 | (3) | 2 | (2) | 18.0 | 6.0 | 8.4 | 18.0 | 6.1 |
| Toe Off | 4 | (8) | 2 | (14) | 3 | (12) | 1 | (3) | 2 | (2) | 18.0 | 11.5 | 13.4 | 18.0 | 9.6 |
| Toe Off 2.5 | 4 | (8) | 2 | (16) | 3 | (14) | 1 | (3) | 2 | (2) | 18.0 | 9.0 | 7.1 | 18.0 | 8.3 |
| WalkOn | 2 | (4) | 2 | (17) | 3 | (13) | 1 | (3) | 2 | (2) | 18.0 | 9.0 | 13.9 | 18.0 | 12.2 |
| WalkOn Plus | 2 | (4) | 2 | (17) | 3 | (12) | 2 | (5) | 2 | (2) | 18.0 | 9.0 | 14.7 | 18.0 | 12.3 |
| Sprystep | 4 | (8) | 1 | (8) | 3 | (14) | 1 | (3) | 2 | (2) | 18.0 | 9.0 | 13.2 | 18.0 | 21.5 |
| Sprystep Max | 4 | (8) | 2 | (17) | 3 | (11) | 1 | (3) | 2 | (2) | 18.0 | 9.0 | 5.6 | 18.0 | 5.1 |
| Sprystep Plus | 4 | (8) | 2 | (23) | 3 | (14) | 2 | (3) | 2 | (2) | 18.0 | 5.5 | 13.5 | 18.0 | 10.9 |
| Matrix | 4 | (8) | 2 | (10) | 3 | (14) | 1 | (3) | 2 | (2) | 18.0 | 18.0 | 9.7 | 18.0 | 20.7 |
| Matrix Max | 4 | (8) | 2 | (12) | 3 | (13) | 1 | (3) | 2 | (2) | 18.0 | 14.5 | 9.9 | 18.0 | 17.8 |
| Matrix Max 2 | 4 | (8) | 2 | (13) | 3 | (14) | 1 | (3) | 2 | (2) | 18.0 | 13.0 | 7.6 | 18.0 | 19.3 |
| Matrix Supermax | 4 | (8) | 2 | (13) | 3 | (11) | 1 | (3) | 2 | (2) | 18.0 | 12.0 | 5.5 | 18.0 | 16.4 |
