# Supplementary figures and images for: Comparison of five different methodologies for evaluating ankle–foot orthosis stiffness
Source: J Neuroeng Rehabil. 2023 Jan 22;20:11. doi: 10.1186/s12984-023-01126-7 (PMC9867850; doi:10.1186/s12984-023-01126-7)

**A**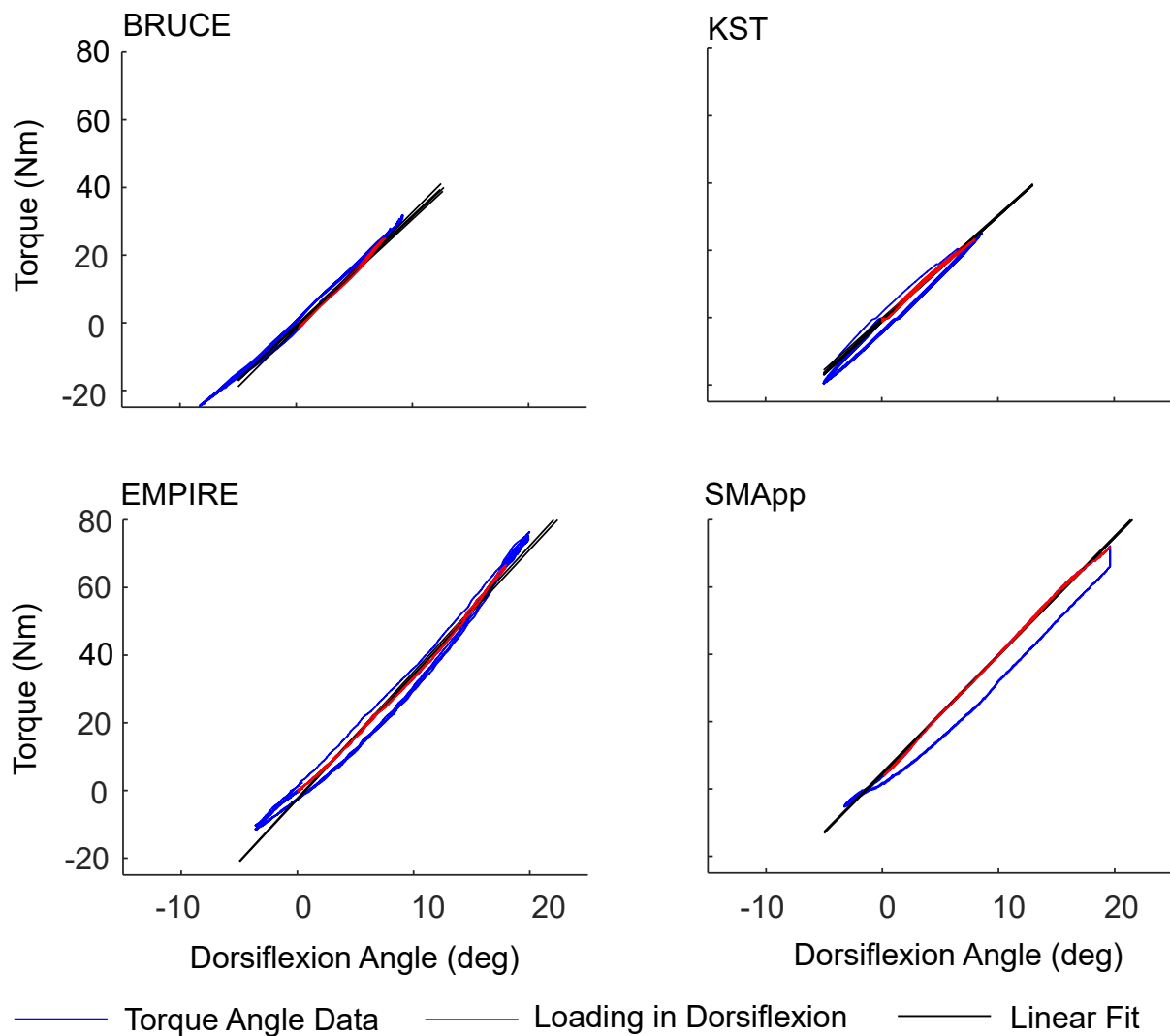**B**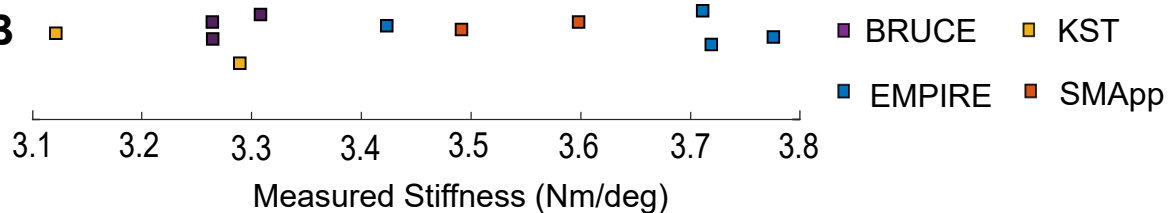

Supplement: Supplementary file 3 — Additional file 3: Figure S1. Blue Rocker. A) Representative test session for each previously described test fixture. AFO stiffness is computed from the linear fit while the AFO is being loaded in dorsiflexion. B) Average stiffness across cycles for each test session for each of the previously described test fixtures. [file 12984_2023_1126_MOESM3_ESM.pdf]

**A**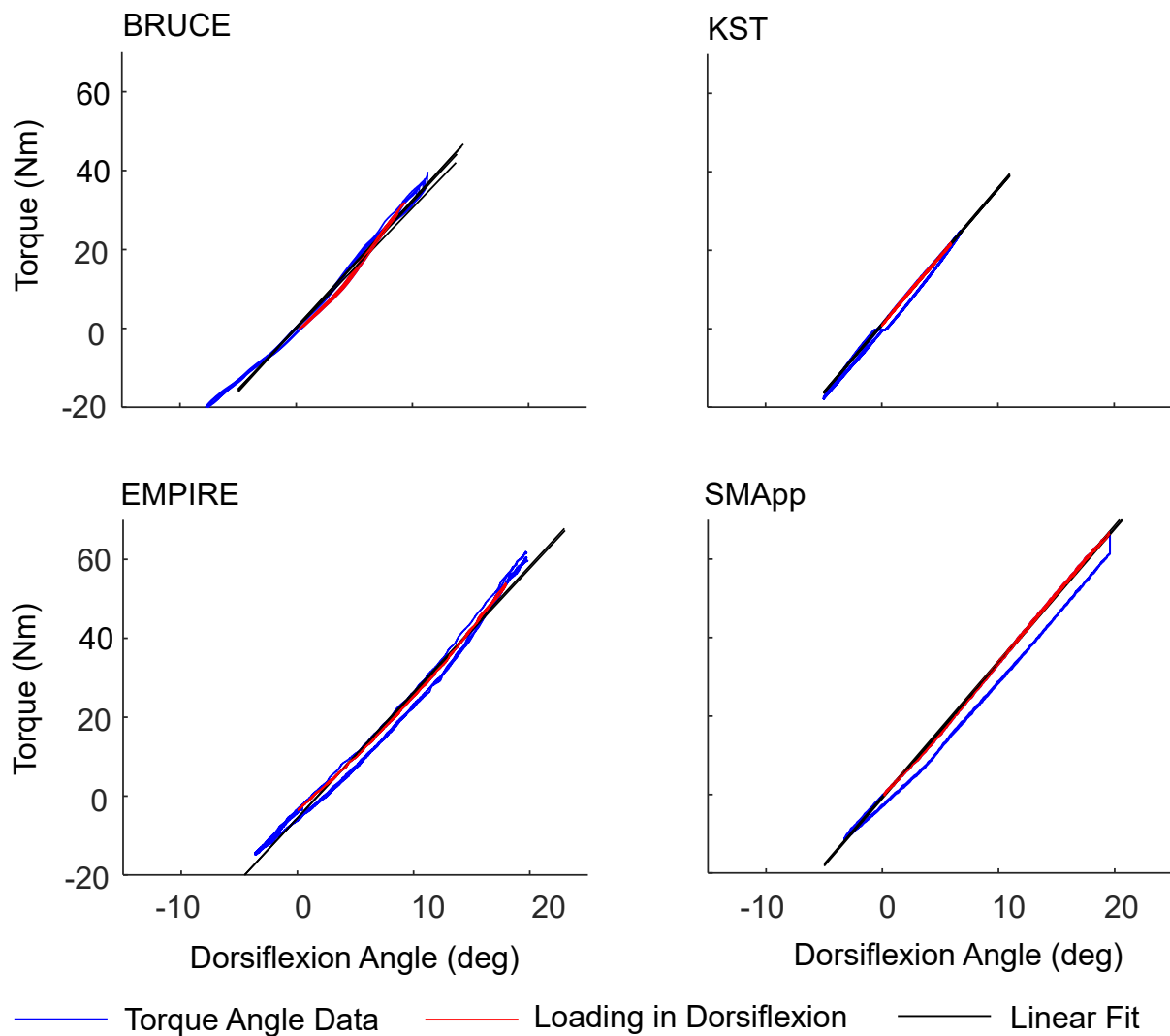**B**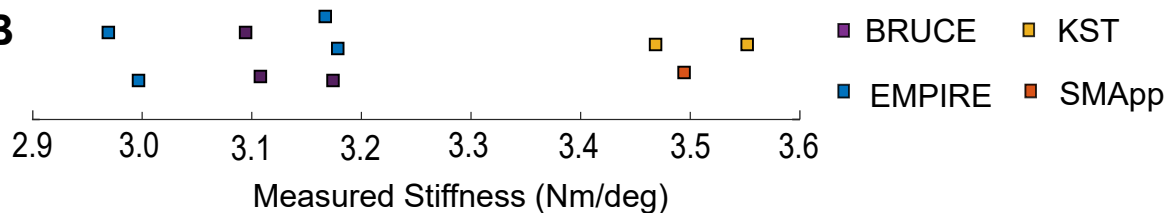

Supplement: Supplementary file 4 — Additional file 4: Figure S2. Blue Rocker 2.5. A) Representative test session for each previously described test fixture. AFO stiffness is computed from the linear fit while the AFO is being loaded in dorsiflexion. B) Average stiffness across cycles for each test session for each of the previously described test fixtures. [file 12984_2023_1126_MOESM4_ESM.pdf]

**A**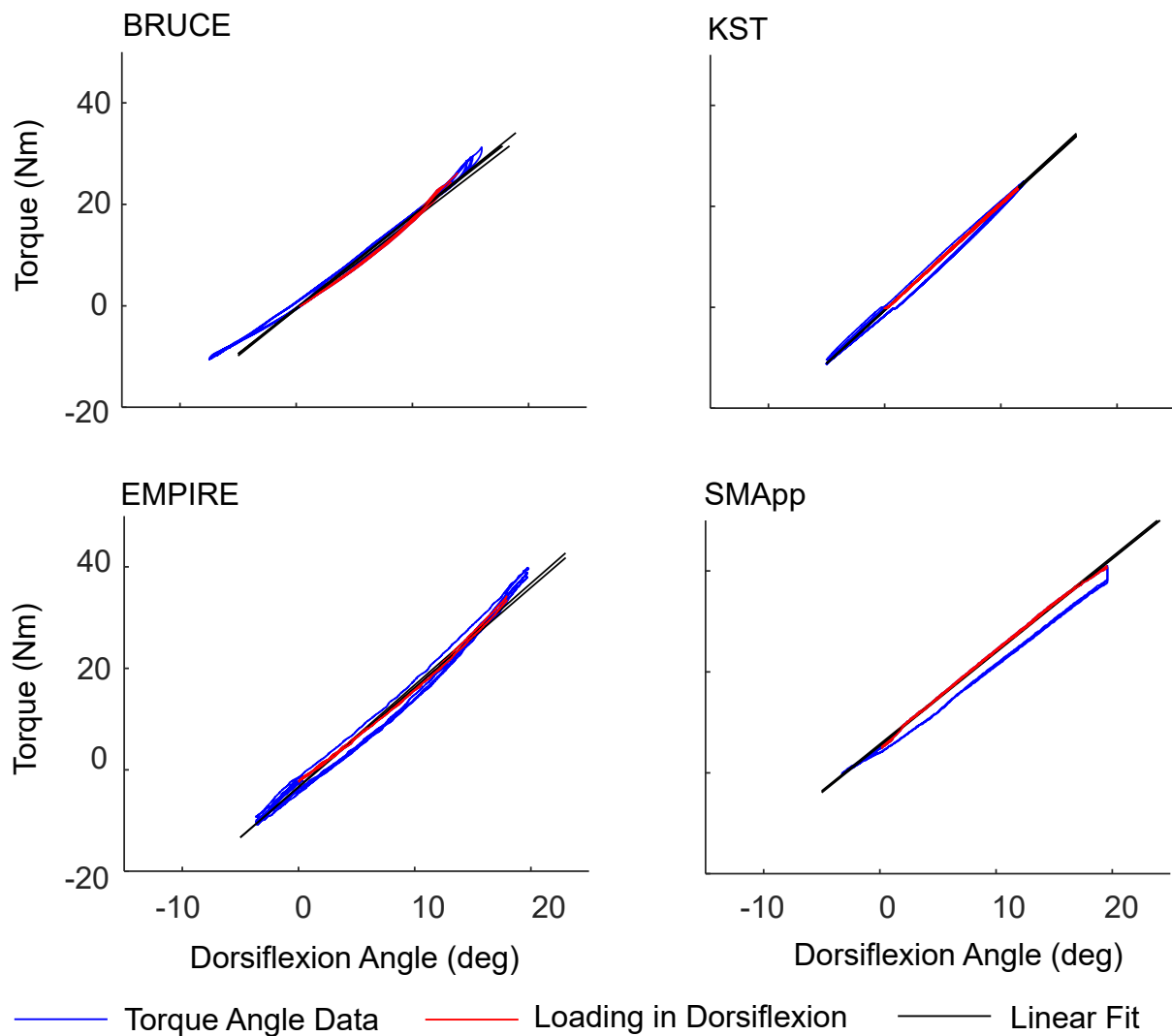**B**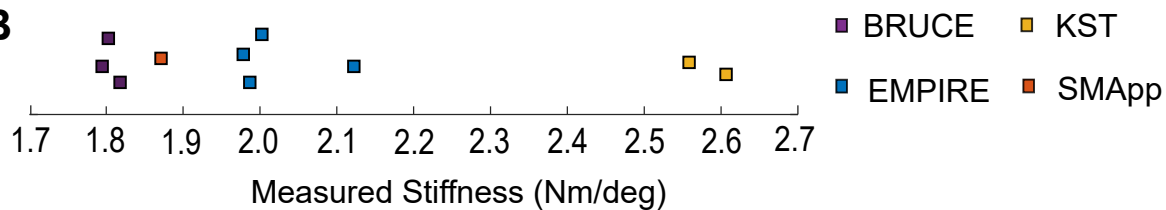

Supplement: Supplementary file 5 — Additional file 5: Figure S3. ToeOff. A) Representative test session for each previously described test fixture. AFO stiffness is computed from the linear fit while the AFO is being loaded in dorsiflexion. B) Average stiffness across cycles for each test session for each of the previously described test fixtures. [file 12984_2023_1126_MOESM5_ESM.pdf]

**A**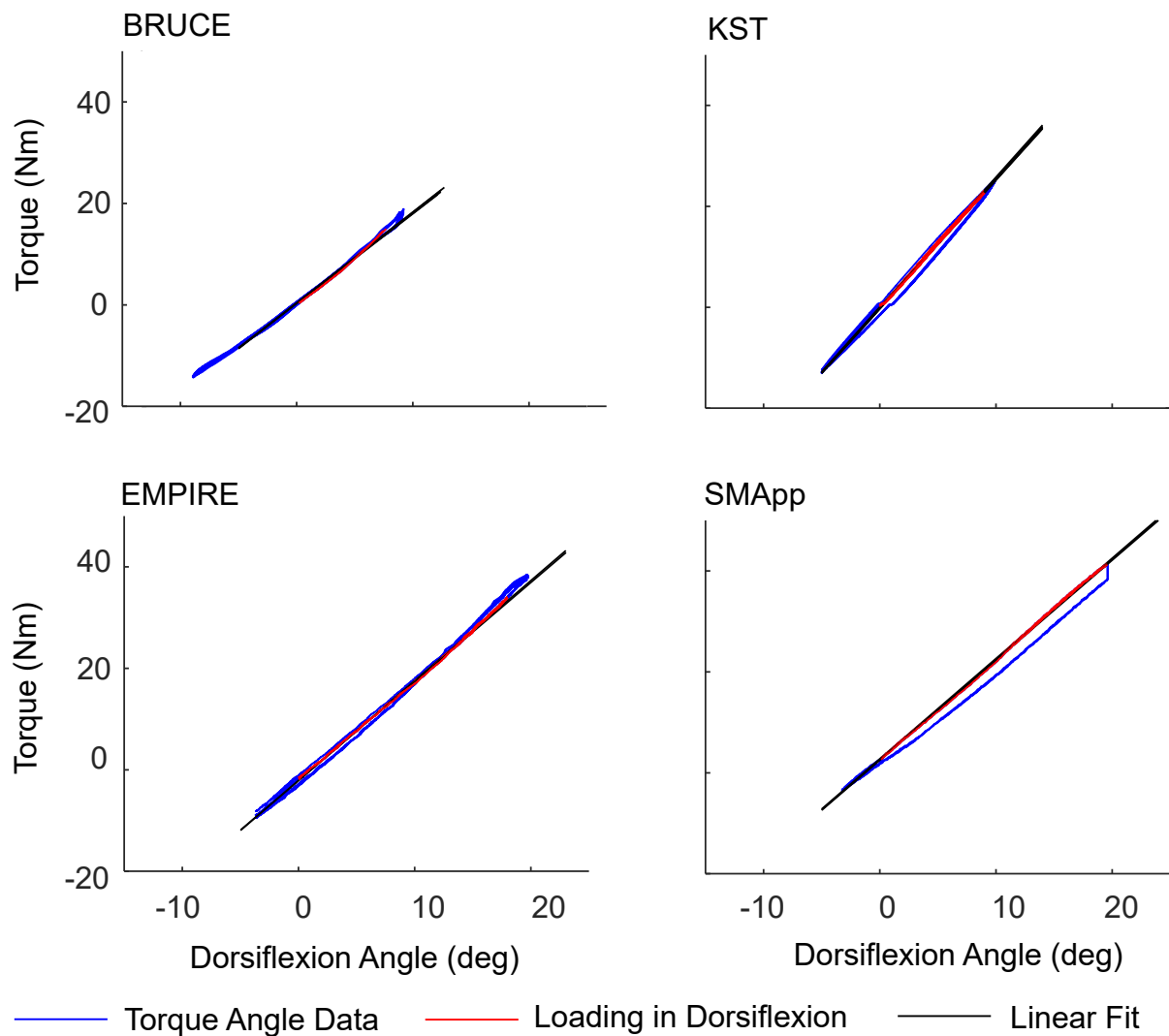**B**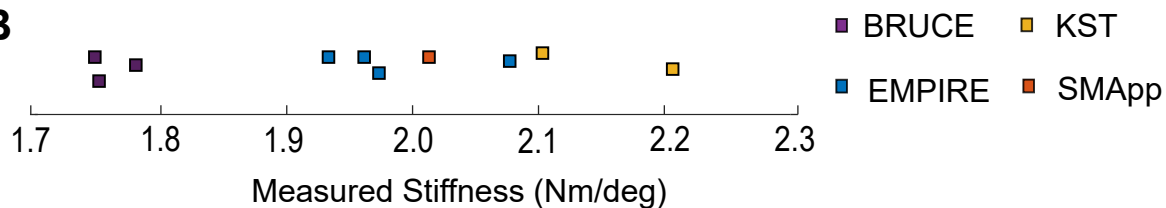

Supplement: Supplementary file 6 — Additional file 6: Figure S4. ToeOff 2.5. A) Representative test session for each previously described test fixture. AFO stiffness is computed from the linear fit while the AFO is being loaded in dorsiflexion. B) Average stiffness across cycles for each test session for each of the previously described test fixtures. [file 12984_2023_1126_MOESM6_ESM.pdf]

**A**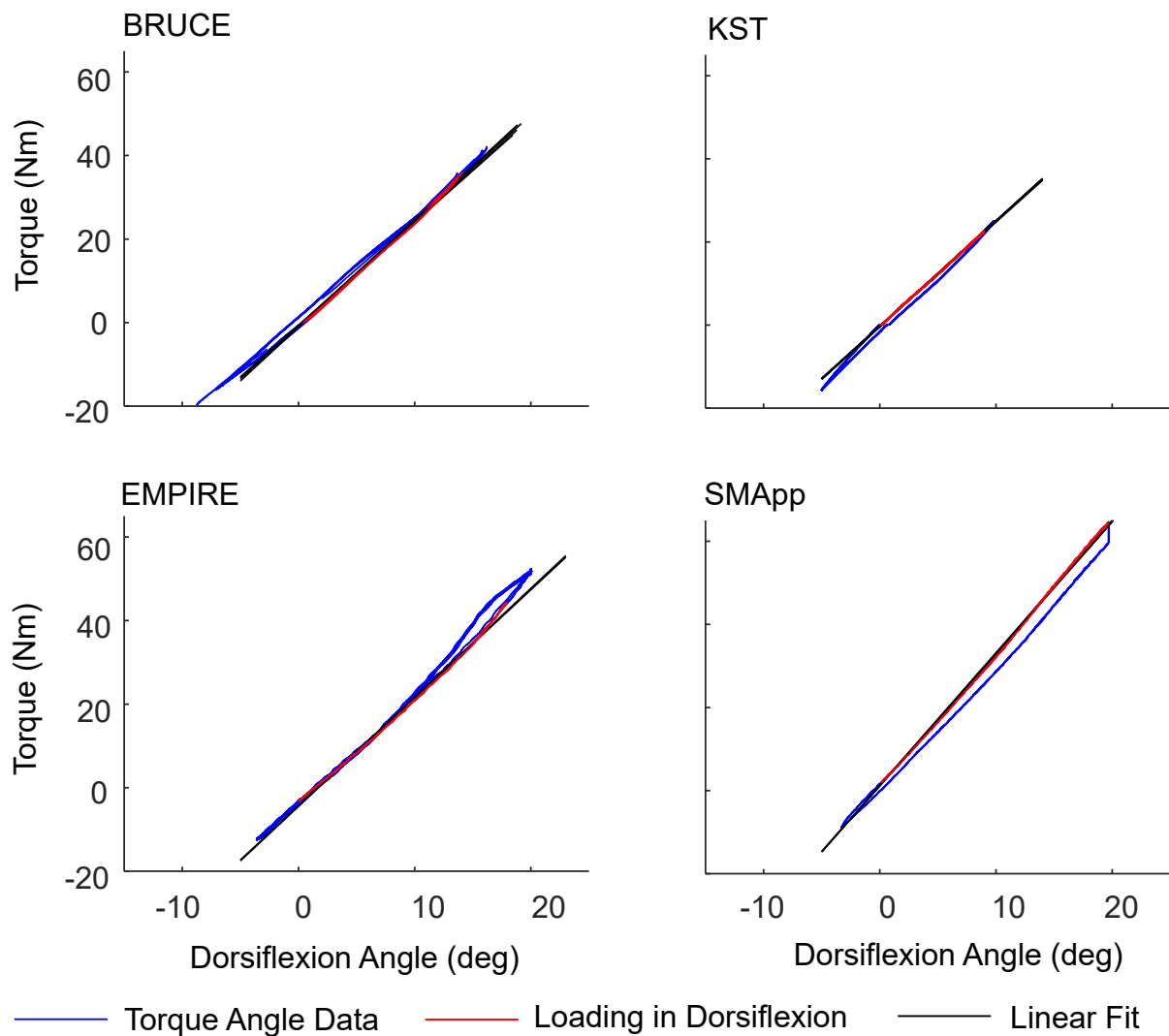**B**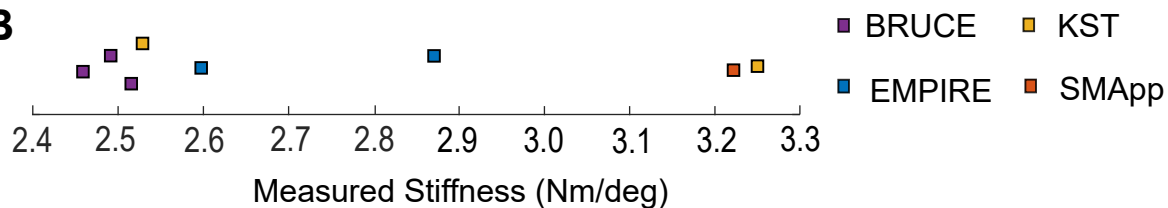

Supplement: Supplementary file 7 — Additional file 7: Figure S5. WalkOn Reaction. A) Representative test session for each previously described test fixture. AFO stiffness is computed from the linear fit while the AFO is being loaded in dorsiflexion. B) Average stiffness across cycles for each test session for each of the previously described test fixtures. [file 12984_2023_1126_MOESM7_ESM.pdf]

**A**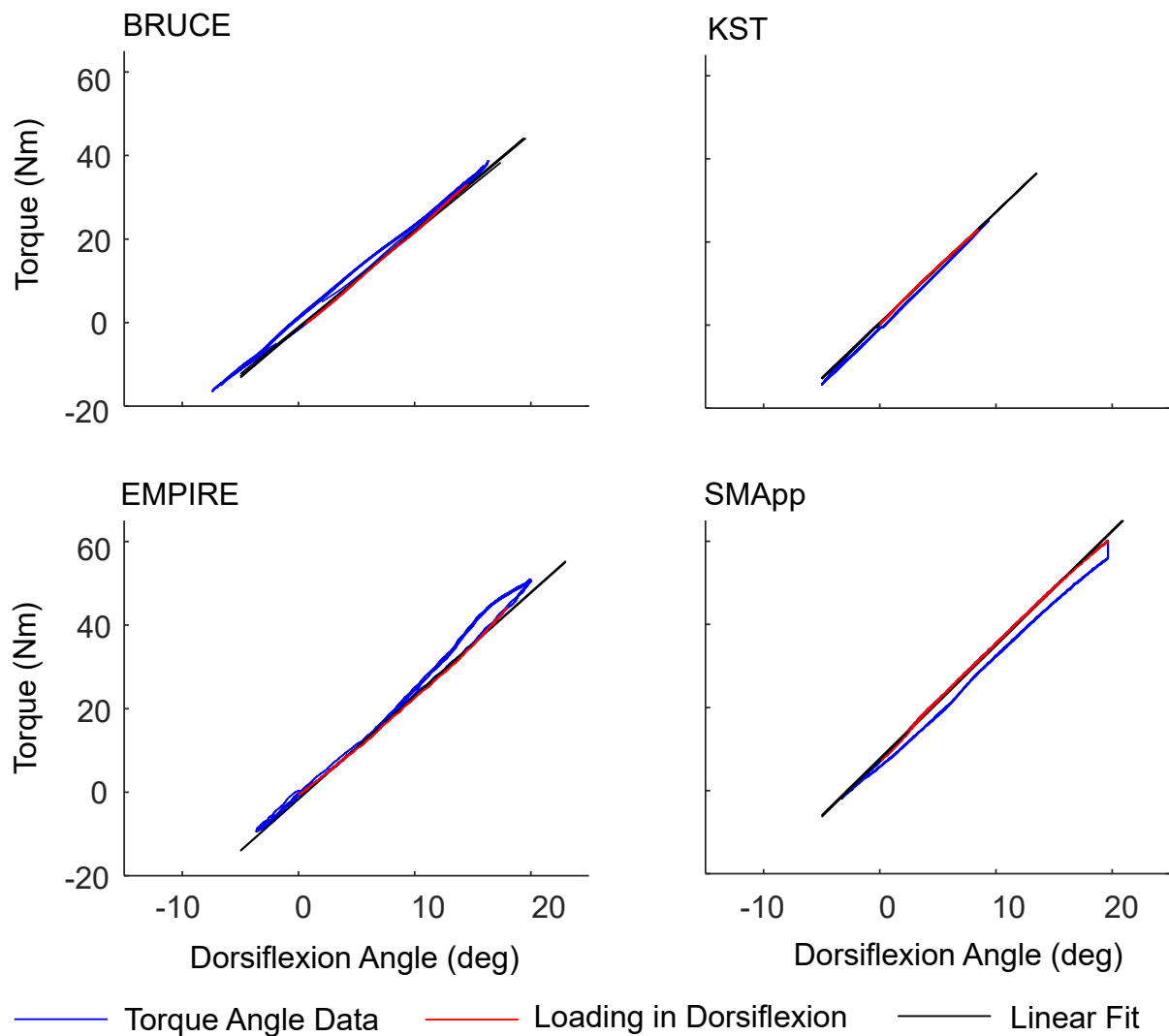**B**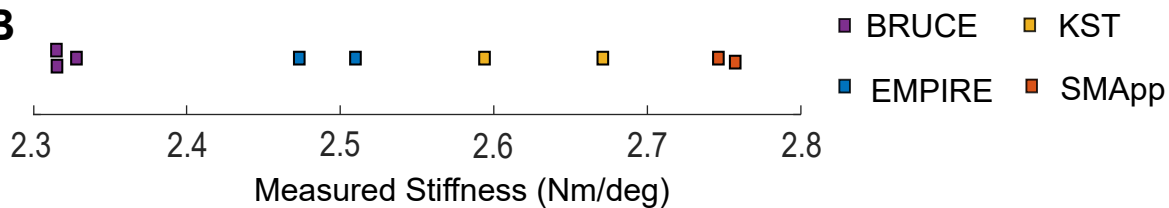

Supplement: Supplementary file 8 — Additional file 8: Figure S6. WalkOn Reaction Plus. A) Representative test session for each previously described test fixture. AFO stiffness is computed from the linear fit while the AFO is being loaded in dorsiflexion. B) Average stiffness across cycles for each test session for each of the previously described test fixtures. [file 12984_2023_1126_MOESM8_ESM.pdf]

**A**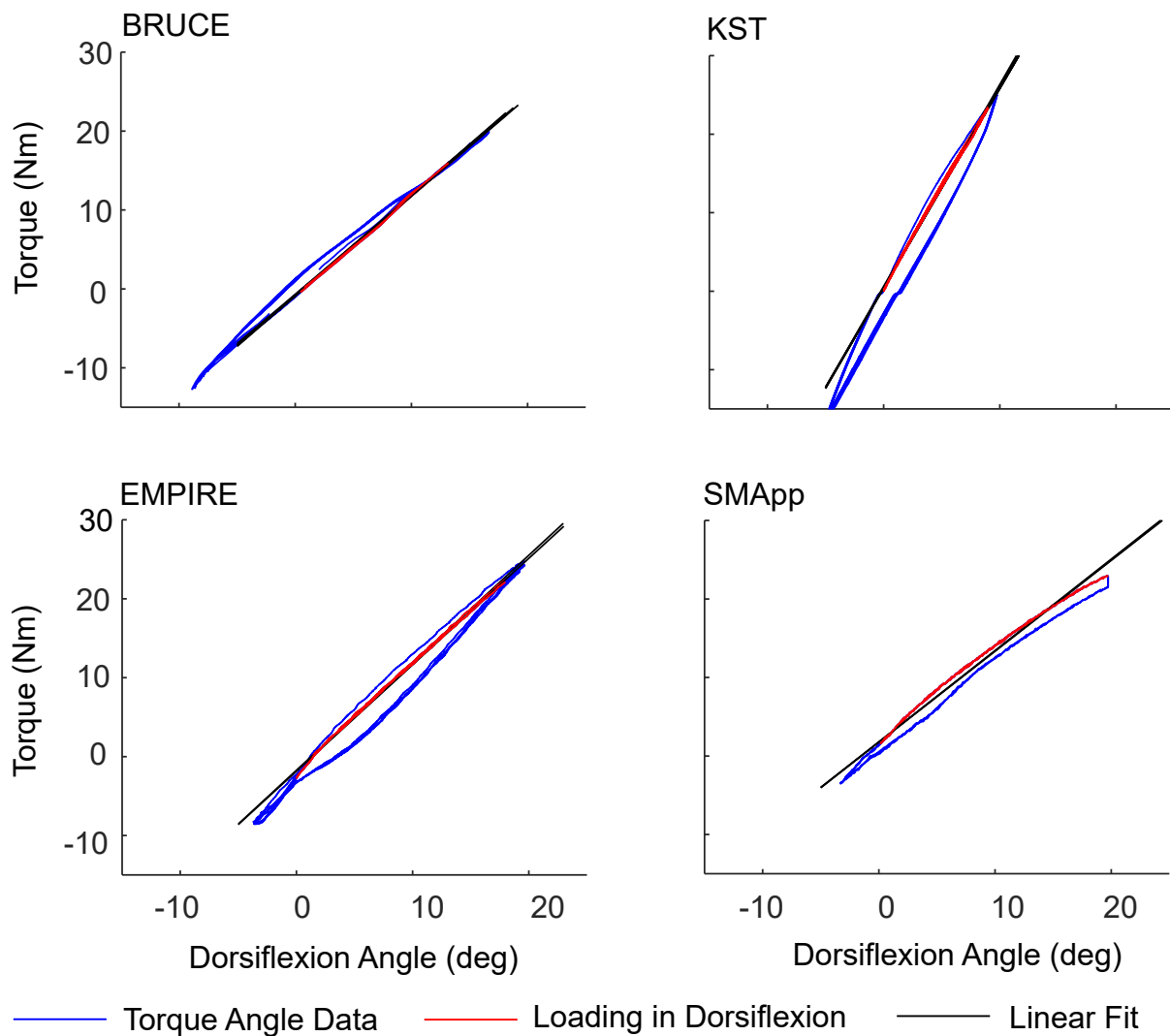**B**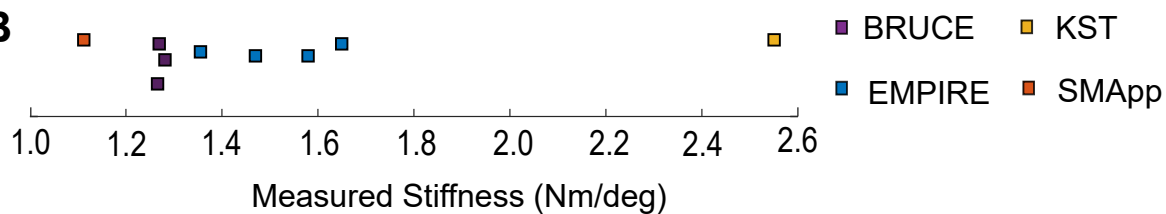

Supplement: Supplementary file 9 — Additional file 9: Figure S7. SpryStep. A) Representative test session for each previously described test fixture. AFO stiffness is computed from the linear fit while the AFO is being loaded in dorsiflexion. B) Average stiffness across cycles for each test session for each of the previously described test fixtures. [file 12984_2023_1126_MOESM9_ESM.pdf]

**A**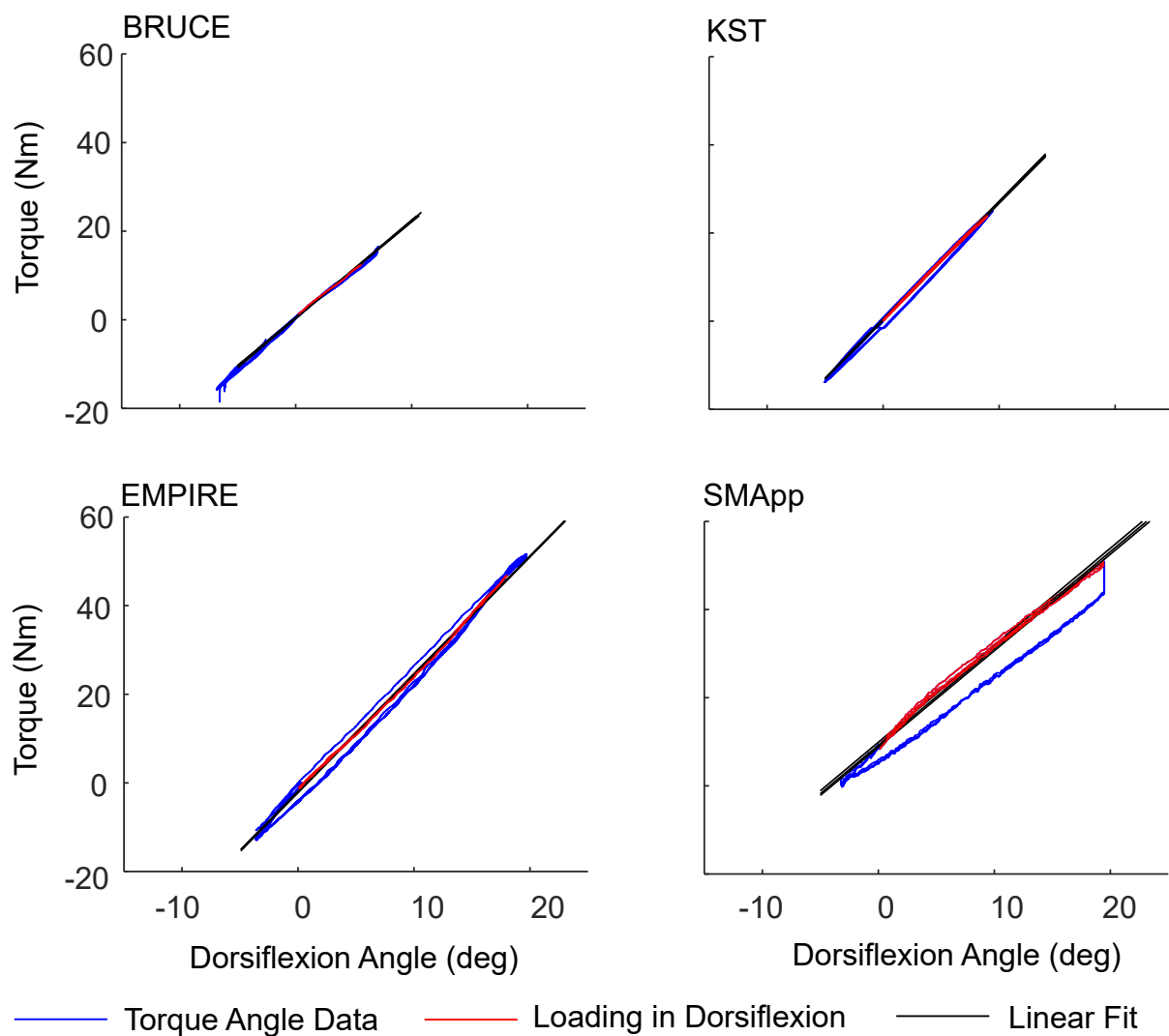**B**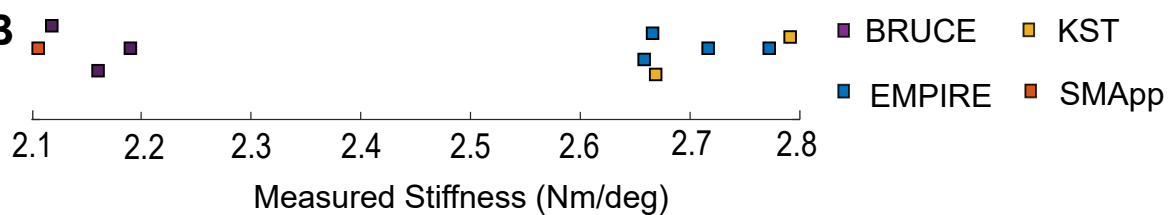

Supplement: Supplementary file 10 — Additional file 10: Figure S8. SpryStep Max. A) Representative test session for each previously described test fixture. AFO stiffness is computed from the linear fit while the AFO is being loaded in dorsiflexion. B) Average stiffness across cycles for each test session for each of the previously described test fixtures. [file 12984_2023_1126_MOESM10_ESM.pdf]

**A**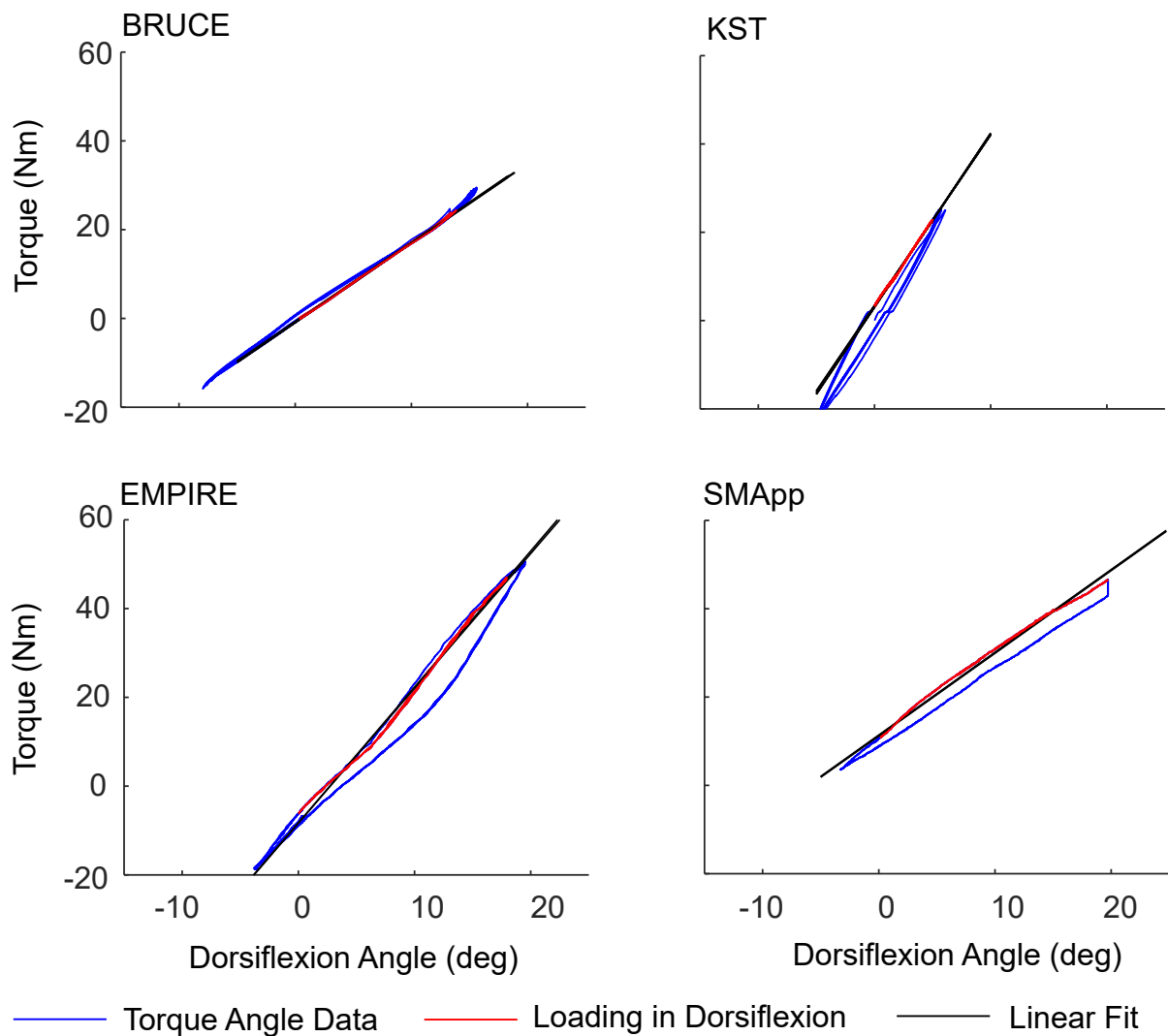**B**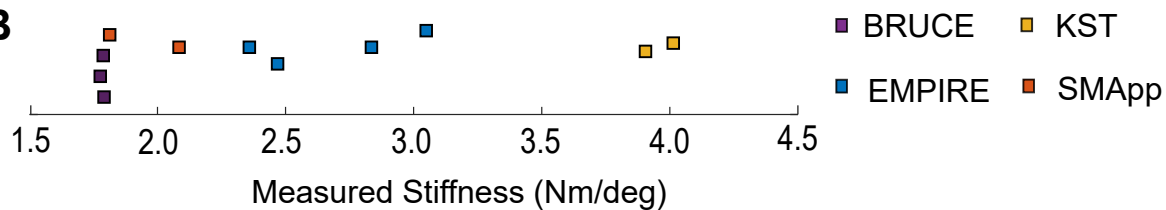

Supplement: Supplementary file 11 — Additional file 11: Figure S9. SpryStep Plus. A) Representative test session for each previously described test fixture. AFO stiffness is computed from the linear fit while the AFO is being loaded in dorsiflexion. B) Average stiffness across cycles for each test session for each of the previously described test fixtures. [file 12984_2023_1126_MOESM11_ESM.pdf]

**A**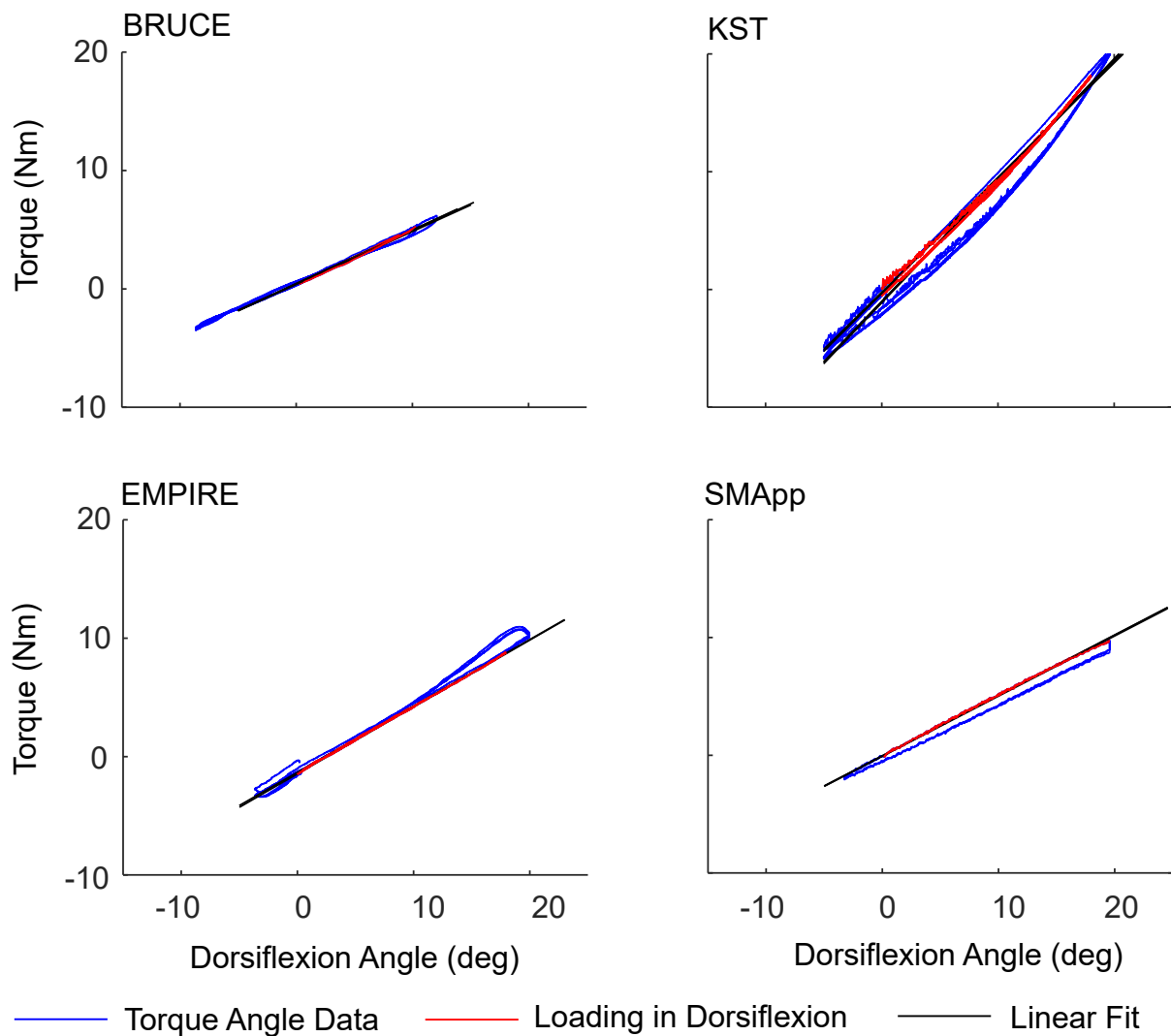**B**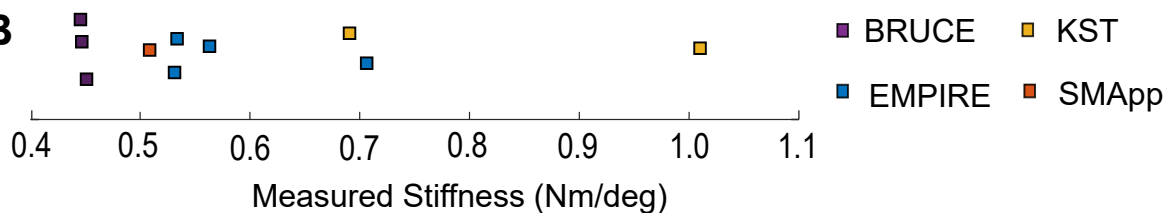

Supplement: Supplementary file 12 — Additional file 12: Figure S10. Matrix. A) Representative test session for each previously described test fixture. AFO stiffness is computed from the linear fit while the AFO is being loaded in dorsiflexion. B) Average stiffness across cycles for each test session for each of the previously described test fixtures. [file 12984_2023_1126_MOESM12_ESM.pdf]

**A**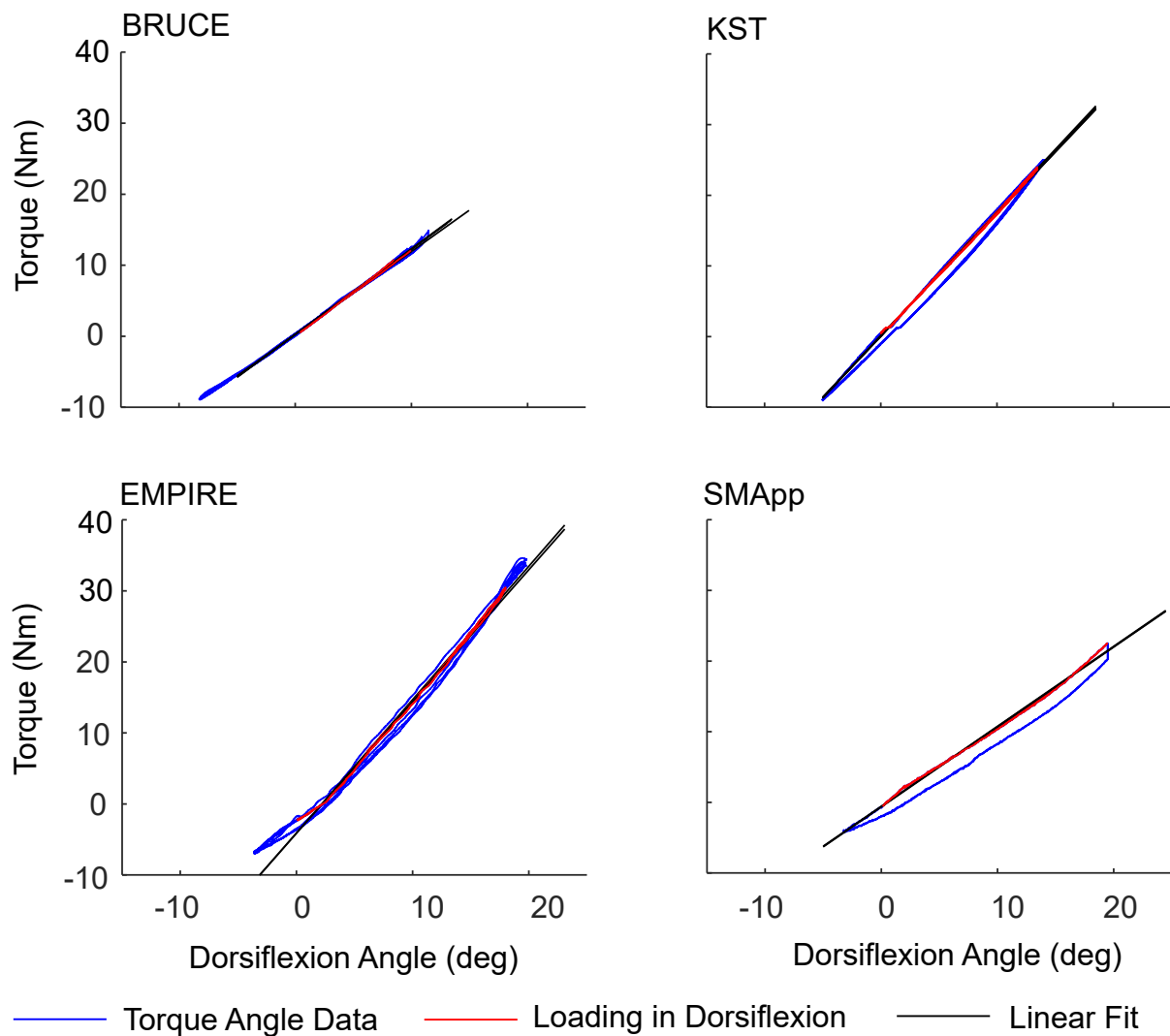**B**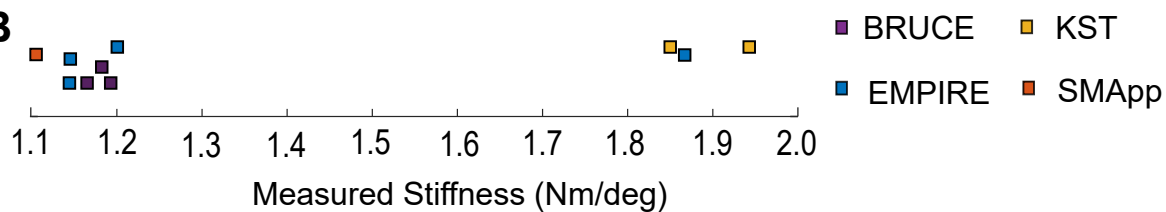

Supplement: Supplementary file 13 — Additional file 13: Figure S11. Matrix Max. A) Representative test session for each previously described test fixture. AFO stiffness is computed from the linear fit while the AFO is being loaded in dorsiflexion. B) Average stiffness across cycles for each test session for each of the previously described test fixtures. [file 12984_2023_1126_MOESM13_ESM.pdf]

**A**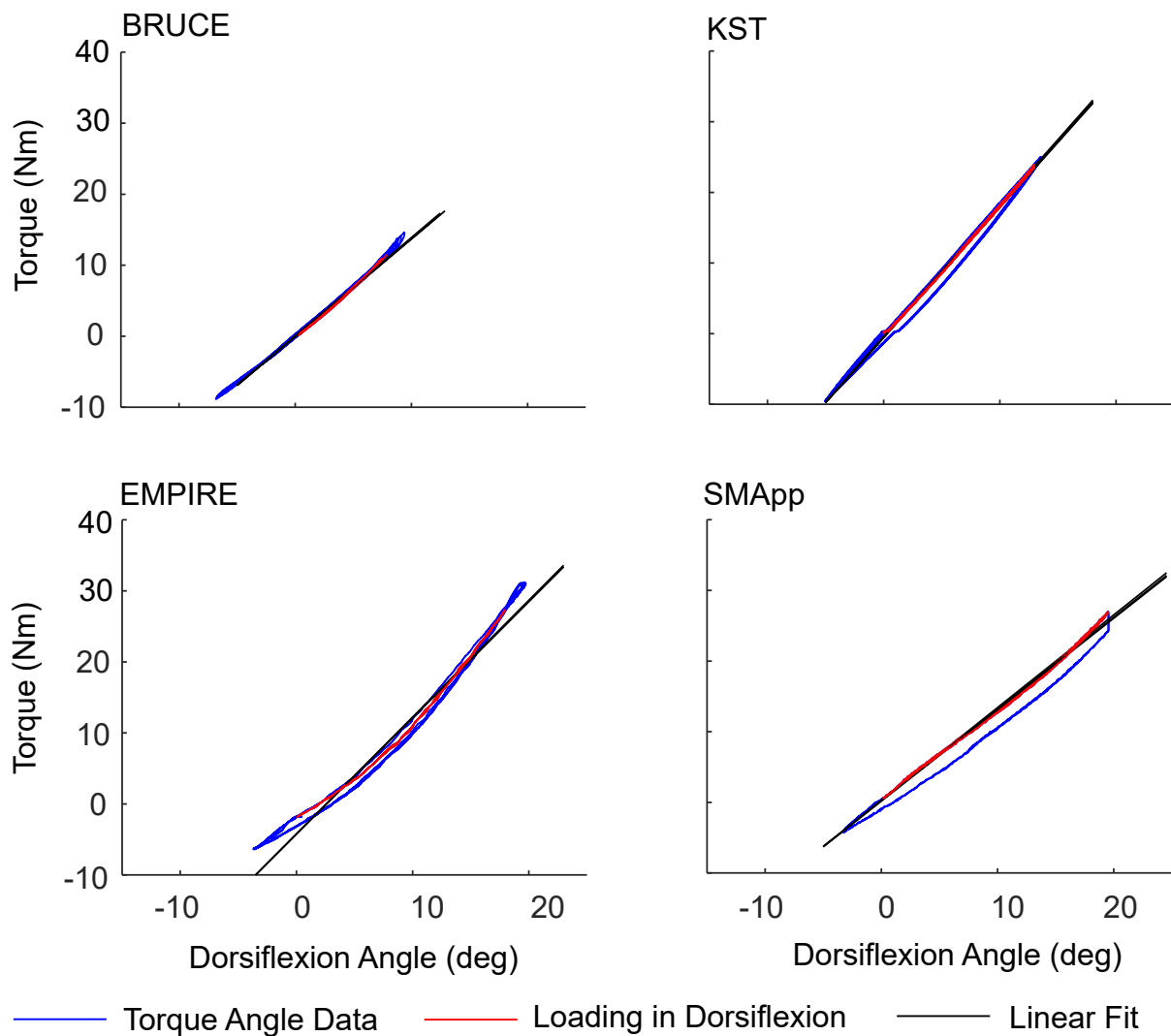**B**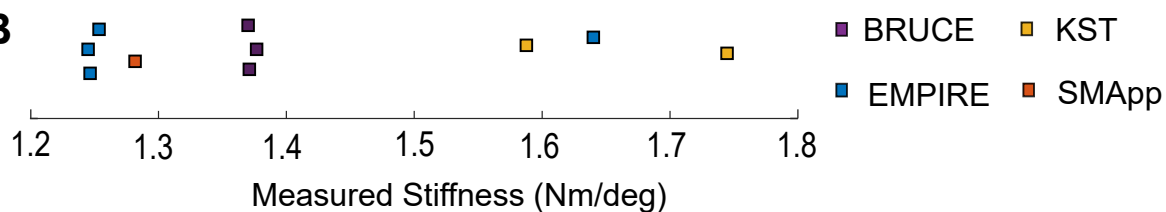

Supplement: Supplementary file 14 — Additional file 14: Figure S12. Matrix Max2. A) Representative test session for each previously described test fixture. AFO stiffness is computed from the linear fit while the AFO is being loaded in dorsiflexion. B) Average stiffness across cycles for each test session for each of the previously described test fixtures. [file 12984_2023_1126_MOESM14_ESM.pdf]

**A**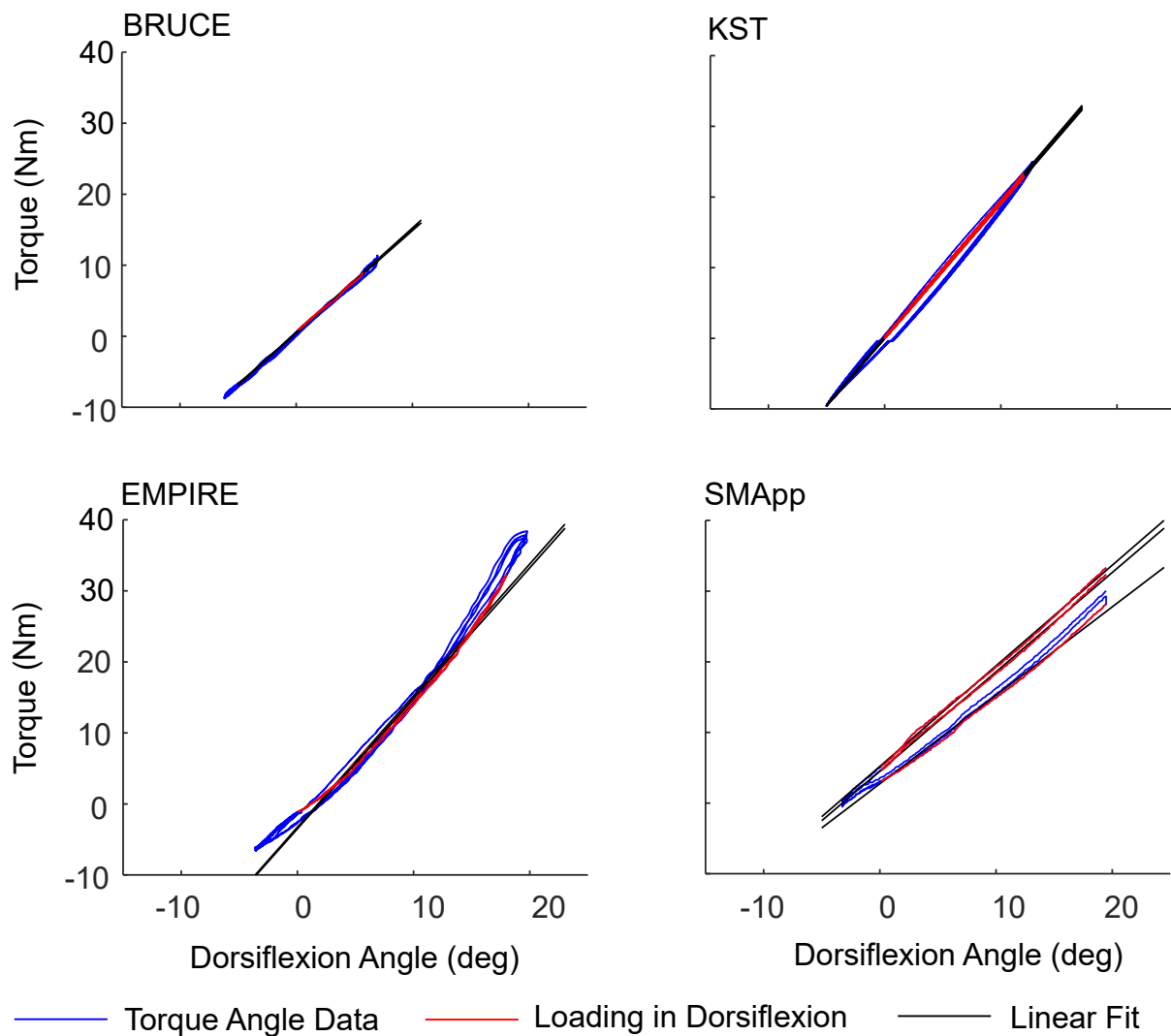**B**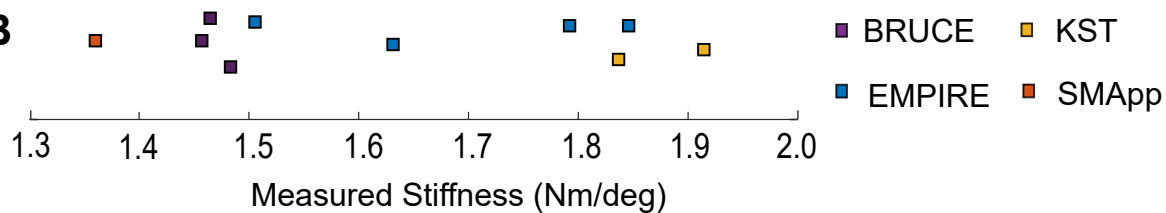

Supplement: Supplementary file 15 — Additional file 15: Figure S13. Matrix SuperMax. A) Representative test session for each previously described test fixture. AFO stiffness is computed from the linear fit while the AFO is being loaded in dorsiflexion. B) Average stiffness across cycles for each test session for each of the previously described test fixtures. [file 12984_2023_1126_MOESM15_ESM.pdf]
